# Effect on Nitrogen Balance, Thermogenesis, Body Composition, Satiety, and Circulating Branched Chain Amino Acid Levels up to One Year after Surgery: Protocol of a Randomized Controlled Trial on Dietary Protein During Surgical Weight Loss

**DOI:** 10.2196/resprot.6438

**Published:** 2016-11-28

**Authors:** Violeta Moizé, Xavier Pi-Sunyer, Josep Vidal, Patricia Miner, Yves Boirie, Blandine Laferrère

**Affiliations:** ^1^ Institut Investigacions Biomèdiques August Pi i Sunyer (IDIBAPS) Barcelona Spain; ^2^ Obesity Research Center Department of Medicine Columbia University New York, NY United States; ^3^ Queens College City University of New York New York, NY United States; ^4^ Unité de Nutrition Humaine Clermont Universite, Université d’Auvergne Clermont-Ferrand France

**Keywords:** bariatric surgery, protein intake, nitrogen balance, body composition, satiety, BCAA

## Abstract

**Background:**

Bariatric surgery (BS), the most effective treatment for severe obesity, typically results in 40-50 kg weight loss in the year following the surgery. Beyond its action on protein metabolism, dietary protein intake (PI) affects satiety, thermogenesis, energy efficiency, and body composition (BC). However, the required amount of PI after surgical weight loss is not known. The current daily PI recommendation for diet-induced weight loss is 0.8 g/kg ideal body weight (IBW) per day, but whether this amount is sufficient to preserve fat-free mass during active surgical weight loss is unknown.

**Objective:**

To evaluate the effect of a 3-month dietary protein supplementation (PS) on nitrogen balance (NB), BC, energy expenditure, and satiety in women undergoing either gastric bypass or vertical sleeve gastrectomy.

**Methods:**

In this randomized prospective study, participants will be randomized to a high protein supplementation group (1.2 g/kg IBW per day) or standard protein supplementation group (0.8 g/kg IBW per day) based on current guidelines. Outcome measures including NB, BC, circulating branched chain amino acids, and satiety, which will be assessed presurgery, and at 3-months and 12-months postsurgery.

**Results:**

To date, no studies have examined the effect of dietary PS after BS. Current guidelines for PI after surgery are based on weak evidence.

**Conclusions:**

The results of this study will contribute to the development of evidence-based data regarding the safe and optimal dietary PI and supplementation after BS.

**Trial Registration:**

Clinicaltrials.gov NCT02269410; http://clinicaltrials.gov/ct2/show/NCT02269410 (Archived by WebCite at http://www.webcitation.org/6m2f2QLeg).

## Introduction

Bariatric surgery (BS) is the most effective long-term therapy for the treatment of severe obesity. BS is associated with a favorable impact on overall and cardiovascular mortality, incidence of first occurrence of fatal or nonfatal cardiovascular events, prevention and remission of type 2 diabetes mellitus (T2DM), and quality of life [1]. Short-term studies showed no apparent difference between gastric bypass (GBP) and vertical sleeve gastrectomy (SG) on T2DM remission and weight loss [2]. GBP and SG are the most accepted procedures currently being performed, with SG increasing in prevalence since its inception in 2003 [3]. Although the surgeries differ widely (GBP is considered a malabsorptive and restrictive procedure while SG is solely restrictive), the prevalence of nutrient deficiencies seems to be comparable [4]. However, the metabolic impact of dietary protein intake (PI) in the early phase of active weight loss has not been studied.

PI during diet-induced weight loss and weight maintenance has been associated with retention of fat-free mass (FFM) [5,6], better satiety [7], and, if insufficient, with malnutrition [8]. Surgical weight loss is associated with decreased circulating levels of branched chain amino acids (BCAAs) [9].

Caloric intake decreases significantly during the first 3-6 months after surgery and may be frequently associated with vitamin, mineral [4,10], and protein deficiencies [4,8-11]. Prospective studies observed that low albumin levels, a clinical marker of protein deficiency [12], can occur up to 2 years after GBP [8,13] with a prevalence ranging from 3 to 18% [9,13-15]. Protein deficiency is more commonly observed after malabsorptive procedures, such as the biliopancreatic diversion [16]. Changes in taste and food preferences, and some degree of stomach discomfort during meals (with or without dumping syndrome), contribute to a poor dietary protein tolerance, thereby affecting the net PI [17]. The potential macronutrient maldigestion and/or malabsorption observed after BS [18] may also contribute to a compromised protein status.

It is generally accepted that diets containing all indispensable amino acids (AAs) are required for optimal protein synthesis and balance [19,20], and optimal intake of dietary protein should even be increased in vulnerable populations [21]. Nitrogen balance (NB), the difference between nitrogen intake and loss, is often compromised with trauma or infection, even with attempted nutritional interventions [22,23]. BS compromises NB via lower PI and an early maintained (or generally higher) protein demand following surgery, so high protein diets are recommended by various guidelines [24-26]. The most updated American Association of Clinical Endocrinologists guidelines suggest that a minimal PI of 60 g/day (and up to 1.5 g/kg ideal body weight [IBW] per day) after BS should be *adequate,* although these recommendations are only supported by a low level of scientific evidence (grade C or D). However, as the clinical tolerance of protein-containing foods is low after BS, recommendations are rarely followed and patients often do not reach their PI goal [17]. Our group and others have shown that daily consumption of 60 g of protein can be challenging during the first 4 months after surgery, even when protein supplements are recommended and supplied at no cost [27]. PI of 1.5 g/kg IBW per day would represent (when considering IBW for a body mass index [BMI] of 25 kg/m^2^) 105 g/day for a woman with an IBW of 70 kg. The low protein tolerance mentioned above makes this recommendation difficult to follow, even with the most motivated patients. Therefore, finding an acceptable amount of PI would ensure optimal FFM retention, limit muscle breakdown, maintain resting energy expenditure (REE), and contribute to the development of a healthier diet that supports weight loss maintenance without interfering with glucose homeostasis.

### Background

#### Risk of Decreased Lean Body Mass and Resting Energy Expenditure With Surgical Weight Loss: Effect of Dietary Protein

The consequences of negative energy and protein balance on visceral mass and skeletal muscle mass are well established [28]. Surgical weight loss results in both fat mass (FM) and lean body mass (LBM) loss: 75.2% and 24.8%, respectively [29]. LBM is the main determinant of REE, and explains 70% of the REE variance [30], with REE being the largest component of 24-hour energy expenditure (EE). Thus, reduced EE after weight loss is a factor of resistance for weight loss, and it may trigger the regaining of weight in the BS population [31]. The impact of daily PI on REE after BS has not been previously addressed, while PI is known to impact postprandial thermogenesis. Although the reduction of FM in obese individuals during weight loss is beneficial, the decrease in LBM may down-regulate the metabolic process, including protein turnover and basal metabolic rate, thus compromising long-term healthy weight management [32]. Studies on the impact of PI on body composition (BC) after BS are scarce and inconclusive. While some studies failed to find a significant correlation between absolute PI and FFM loss relative to total weight loss after BS [5,33-35], others found that higher levels of PI improved BC changes by enhancing the loss of FM and reducing FFM loss after BS [36]. High protein diets may increase EE while preventing LBM loss [30] during weight loss. Increased EE from dietary protein is attributed to an enhanced thermic effect of food (15%, standard deviation [SD] 4) compared to carbohydrates (6%, SD 2) or lipids (7%, SD 3) [37]. Studies related to the effect of high protein versus standard protein diets on the prevention of LBM loss, which in turn lead to a lesser reduction in REE, are often inconclusive [8,34,38]. In addition, comparisons between GBP and SG have not been completed.

#### Nitrogen Balance Study in the Bariatric Surgery Setting

NB is classically used to determine adequate PI with regards to daily nitrogen loss, and to estimate whole body protein balance in response to nutritional interventions [39]. Sustained negative NB can be associated with loss of lean and fat tissue [40]. Thus, ideally the goal of PI after BS should aim at preventing and/or limiting negative NB, even under energy restriction.

Occurrence of malabsorption should be considered when assessing NB in BS subjects [11]. In a malabsorptive state, fecal losses of nitrogen may be as high as 3.5 g/day [12]. Thus, in addition to the other components of the NB equation, fecal nitrogen losses should be measured after BS, and not simply estimated at 0.4 g/day, as recommended by the Joint Food and Agriculture Organization/World Health Organization expert committee, under nonmalabsorptive conditions [41,42]. Of note, Odstrcil et al [18] studied the contribution of malabsorption on the reduction in net energy absorption 5 and 14 months after long-limb GBP. Net absorption of protein was significantly reduced after BS, and malabsorption accounted for 13% of the total reduction in protein absorption at both study time points [18]. However, a protein kinetic study using stable isotopes demonstrated that protein digestion and absorption were not impaired, and even accelerated, 3 months after GBP [43].

#### Roles of Protein Supplementation on Circulating Levels of Amino Acids

It has long been recognized that circulating levels of AAs, including BCAAs, are elevated in persons with obesity, insulin resistance (IR), or T2DM, compared to healthy controls [44,45]. BS is associated with reduced concentrations of plasma BCAAs [46] and decreased IR [8,46]. To date, the protein sparing effect of long-term protein supplementation (PS) has not been studied.

#### Dietary Protein Intake and Satiety

High PI has been shown to increase satiety in the context of energy restriction [47,48]. Proposed factors that may enhance satiety include: a ketogenic state; relatively elevated plasma AA levels [49]; an increase of the satiety peptide YY (PYY), glucagon-like peptide 1 (GLP-1) and cholecystokinin (CCK) [50]; and/or a decrease of the orexigenic hormone ghrelin [13,51]. We aim to further explore the relationship between PI and satiety after BS procedures.

### Study Aims

Considering the complexity of metabolic and behavioral changes after BS, the overall aim of our research study will be to establish adequate PI after BS. To achieve this goal, we will compare the effect of 2 levels of PI (high protein supplementation group, HPS-G; and standard protein supplementation group, SPS-G) after BS (GBP and SG) on (1) NB, (2) BC, (3) REE and diet-induced thermogenesis (DIT), (4) satiety, (5) the release of gut hormones, (6) circulating levels of BCAAs in relation to insulin sensitivity, and (7) adherence to protein supplements. Five specific objectives will address our aims.

#### Objective 1: Nitrogen Balance

Total body NB will be measured to assess the levels of PI and protein absorption. The measure of NB will be performed during an inpatient stay before surgery, after 3 months of controlled PS, and 12 months after BS. PI will be established at 1.2 g protein/IBW per day for all participants in the month before surgery. After surgery, participants will be randomized to either 1.2 g protein/IBW per day (HPS-G) or 0.8 g protein/IBW per day (SPS-G). Participants will receive PS for 3 months after BS, up to the second inpatient study time point. During the inpatient stay, all foods and drinks will be strictly controlled, and 24-hour urine and stool samples will be collected. Nitrogen content of food from aliquots, urine, and stool will be measured, as explained in the *Methods* section.

#### Objective 2: Effect of Protein Supplementation on Body Composition

We will compare the effect of HPS and SPS on LBM. Changes in BC will be assessed before surgery, at 3 months, and 12 months after surgery in the 2 PS study groups using anthropometry, total body water (TBW), and plethysmography (BOD POD).

#### Objective 3: Effect of Protein Supplementation on Resting Energy Expenditure and Diet-Induced Thermogenesis

We will compare the effect of HPS and SPS on EE, measured by indirect calorimetry at rest (REE) and 4 hours after a high protein liquid test meal (DIT), before surgery, at 3 months, and 12 months after BS.

#### Objective 4: Branched Chain Amino Acid Levels

We will characterize changes in circulating BCAA levels in relation to insulin sensitivity and PI adequacy after GBP and SG. Circulating BCAA levels will be measured by targeted metabolomics and compared to insulin sensitivity (calculated by the homeostatic model assessment-insulin resistance or Matsuda index) before surgery, at 3 months, and 12 months postsurgery.

#### Objective 5: Effect of Surgical Procedures on Nitrogen Balance and Satiety

We will compare the effect of HPS and SPS, the hormonal response after a meal, and NB between GBP and SG. Satiety and hunger will be measured by visual analog scales (VASs) while fasting and in response to a high protein meal, before surgery, at 3 months, and 12 months after BS. Blood samples will be obtained before and after the meal test to measure the satiety-related gut hormones CCK, PYY, GLP-1, ghrelin, along with insulin and glucose levels. NB will also be compared between the surgical procedures.

## Methods

### Subjects

All subjects will be recruited from the Bariatric Surgery Institute at Mount Sinai St Luke’s Hospital (New York, NY), and will be required to sign an institutional review board -approved consent form prior to enrollment.

#### Number of Subjects

A total of 112 volunteers scheduled to undergo either GBP or SG will be recruited. Based on our experience, we anticipate a conservative 30% attrition rate. Therefore, approximately 80 patients are expected to remain in the study at completion.

#### Inclusion and Exclusion Criteria

BC differences exist between women and men, and the BS patient population is 75% women, so only premenopausal women (18-40 years of age, BMI <50 kg/m^2^) will be included in this study. Other inclusion criteria include: any race/ethnicity; patients scheduled to undergo either GBP or SG; treated or untreated resting systolic/diastolic blood pressure less than 160/100 mmHg; fasting triglyceride concentration less than 600 mg/dl, without regard for diagnosis or prescription for other dyslipidemias; absence of diabetes, or diet-controlled diabetes (taking no medications).

Exclusion criteria include: presence of nitrogen retention disease (eg, renal or hepatic disease); abnormal thyroid function; known malabsorption syndrome; cardiovascular disease; current mucosal (gastrointestinal, respiratory, urogenital) or skin (cellulitis) infection; any psychiatric disorder; and any other condition which, in the opinion of the investigators, may make the candidate unsuitable for participation in this study.

### Study Design

#### Design and Setting

**Figure 1:**
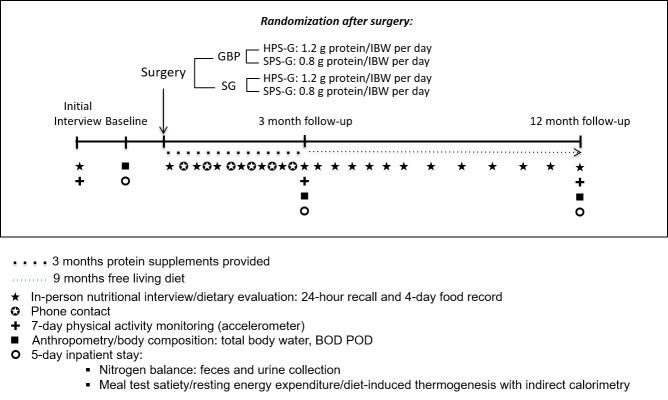
Overall study design of protein supplementation after bariatric surgery. GBP: gastric bypass; SG: sleeve gastrectomy; HPS-G: high protein supplementation group; SPS-G: standard protein supplementation group; IBW: ideal body weight.

This study is a prospective randomized controlled trial (RCT), in which 112 obese participants (with no major comorbidities) scheduled to undergo GBP or SG will be randomly allocated to SPS-G or HPS-G cohorts. Participants will undergo three 5-day inpatient stays: presurgery, 3 months postsurgery, and 12 months postsurgery. The inpatient stays will be in the Clinical Research Resource (CRR) in the Irving Institute of Clinical and Translational Research at Columbia University Medical Center. The overall study design is displayed in Figure 1.

#### Protein Supplementation Protocol

PS regimens will be supplied during the 3 months immediately following surgery. The HPS-G group will receive 1.2 g protein/kg IBW per day, and the SPS-G group will receive 0.8 g protein/IBW per day, with IBW established as a BMI of 25 kg/m^2^ [4]. PS goals will be achieved partly by providing subjects with an instant high quality protein-isolate whey protein powder-supplement (Unjury; ProSynthesis Laboratories, Sterling, VA) [52]. Whey protein is the richest source of BCAAs and leucine, which has been demonstrated to stimulate muscle protein synthesis in elderly populations [53,54]. The SPS-G group will require approximately 1.5 servings per day (21 g protein/serving) while the HPS-G group will require 3 servings per day to meet their individually prescribed protein requirement.

#### Preceding Diet at Baseline

The NB study will be preceded by an outpatient diet stabilization period lasting 7 days. During this time, a meal plan will be tailored to each participant and PS will be provided, based on the individual’s preferences, to promote adherence. Participants will be asked to complete a 7-day food record during this outpatient period, and will be contacted by phone one time during this period in order to evaluate adherence to, and tolerance of, the prescribed diet. This diet stabilization period is designed to allow each participant to adapt to the required level of PS [55]. Energy and protein requirements will be based on 35 kilocalorie (kcal)/IBW per day and 1.2 g protein/IBW per day, respectively [56], and will be calculated using the Nutrition Data System for Research (NDSR), 2011 [57]. Energy intake will be adjusted to minimize weight changes (within 0.75 kg of body weight) during the period of controlled diet, prior to admission, and during the inpatient stay [58].

#### Inpatient Study Period Before Surgery

During the initial 5-day inpatient study period all foods and drinks will be provided, and carefully monitored and controlled, under the supervision of the research dietician (VM) and the staff of the Bionutrition Unit in the CRR. All foods, drinks, and protein supplements will be aliquoted and weighed on a 0.1-gram precision scale before consumption. To minimize variability and accurately control the intake of nitrogen, participants will eat the same diet every day. Participants will not be allowed to eat or drink any other food except for tap-filtered water. Diets will be based on regular/natural foods that require minimum manipulation. To meet the dietary PI goals, Unjury isolate whey protein products will be included in the diet as the main source of protein during the entire 5-day period. Vitamin and mineral supplementation, if any, will be continued during the inpatient study. Participants will be asked to eat the entire food portion that is served. Supervision will be ensured during each meal, and after each serving the trays will be examined by staff members, and the volume of any unconsumed beverage/soup will be measured in a graduated cylinder. All existing uneaten food will be weighed and recorded for day-to-day dietary adjustments. Meals for 1 complete day during the stay will be prepared and cooked in duplicate, and will be homogenized in a heavy duty commercial blender to obtain 24-hour diet aliquots. Two aliquots will be frozen at –70° Celsius for later analysis of total nitrogen and energy content.

#### Post-Bariatric Surgery Dietary Protein Supplementation Intervention

After BS, participants will be randomized using the technique of permuted block randomization to ensure that equal numbers of patients are assigned to each treatment arm (HPS-G or SPS-G). In accordance with post-BS practice guidelines, the meal plan will consist of 6 small meals per day (breakfast, morning snack, lunch, afternoon snack, dinner, and late snack), plus powdered PS that will be distributed during either 3 (SPS-G) or 6 (HPS-G) meals. Phone and in-person contacts will be provided on alternating weeks during the 12-week PS intervention. At each contact time point, dietary and PS adherence, food tolerance, and hydration will be evaluated by reviewing the food records specifically designed to quantify the number of scoops of the PS used in each meal. Urinary nitrogen excretion will be used as a biomarker for PI, and to measure compliance. Subjects will be asked to collect their 24-hour urine at 5 different time points during the 12-week period. PS containers will be supplied during the in-person participant-dietitian biweekly interviews. Participants will be asked to bring the empty PS containers for review and quantification at each appointment.

#### Preceding Diet and 5-Day Inpatient Stays at 3 Months and 12 Months After Bariatric Surgery

Daily energy intake during the preceding diet period, and during the inpatient stays, will be significantly reduced to a 10-15 kcal/IBW per day at 3 months post-BS. This value will increase up to 20 kcal/day at 12 months [4].

### Laboratory Analyses and Outcome Measurements

#### Nitrogen Balance Study

Participants will be admitted for 5 days in the CRR for complete feces and urine collections during the last 3 days of the inpatient period. These collections will take place at the 3 different study time points: (1) presurgery; (2) 3 months after BS, during the active weight loss phase; and (3) 12 months after BS to evaluate the long-term carry over effect of PS on BC changes and REE.

#### 24-Hour Complete Specimens Collection

Four 24-hour urine collections will be undertaken; 1 per day during the inpatient stay. Collection will start after the first void of the second day. Labeled urine containers will be refrigerated at 4° Celsius. Starting on the third day, and until the end of the inpatient study period, all stools will be collected with a specimen container kit placed in the toilet. Samples will be processed every day and aliquots will be obtained and stored at –70° Celsius for later analysis of 24-hour nitrogen content. To ensure a complete collection of feces and compliance with the inpatient diet, 1.5 g of polyethylene glycol (PEG) will be ingested along with other food items during the first inpatient day. PEG has been used as a fecal marker to follow time and completeness of collections, to determine when experimental diets have been eliminated, and to correct for differences in the day-to-day variation of fecal transit time [59,60]. Stool samples will be analyzed for PEG, and combustible energy from PEG will be subtracted from fecal combustible energy measured by bomb calorimetry.

Miscellaneous losses of nitrogen in sweat, sloughed skin, nails, hair, and various bodily secretions will be estimated at 8 mg/kg of body weight per day [42]. Body weight will be measured daily during the inpatient stays to estimate the daily miscellaneous nitrogen losses defined above.

NB will be calculated using the difference between daily nitrogen intake and total nitrogen losses as follows: nitrogen balance = dietary nitrogen intake–(daily urinary nitrogen excretion + daily fecal nitrogen excretion + daily miscellaneous nitrogen losses).

#### Anthropometry and Body Composition

##### Anthropometry

Anthropometry will be assessed during admission at the CRR before BS, at 3 months, and 12 months after surgery. Participants will be weighed while wearing a hospital gown, and without shoes, to the nearest 0.1 kg. Height will be determined using a fixed wall stadiometer to the nearest 0.1 cm. BMI will be calculated as weight (kg) divided by the square of the height (meters).

##### Body Composition

BC will be estimated based on a 3-compartment model, using 3 independent measures: body weight, TBW, and body density. To determine changes in BC after BS-induced weight loss, the 3-compartment model will be used [61].

##### Total Body Water

TBW will be measured using the stable isotope deuterium oxide (D_2_ O) as the reference method. A baseline venous blood sample (approximately 7 mL) from an antecubital vein will be taken while fasting. Immediately afterwards, a known dose of deuterated water D_2_ O (1 g/kg) will be taken orally. Three hours following the dose, when the D_2_ O has equilibrated with the body water deuterium-to-hydrogen, a second fasting blood specimen will be taken (approximately 7 mL). The TBW will then be derived from the increase in plasma deuterium content in relation to the volume of D_2_ O ingested [62].

##### Air Displacement Plethysmography

Body density will be measured using the BOD POD (Cosmed, Chicago, IL; software version 2.3) [63,64]. Subjects will be clothed in a Lycra-style swim cap and tight fitting underwear. Body weight will be measured to the nearest 1 g on the BOD POD electronic weight scale. Following standard calibration procedures, the subject’s body volume will be measured, with correction made for thoracic gas volume, which will be estimated using the BOD POD breathing circuit system. The final thoracic gas volume and the average of 2 body volume measurements within 0.2% will be used to calculate body density [64].

##### Three-Compartment Calculations

FFM and FM will be measured using a modified 3-compartment model that was developed for obese subjects in the New York Obesity Research Center Body Composition laboratory, as follows: fat (kg) = 2.122 × (body weight/density) – 0.779 × TBW – 1.356 × body weight [65]. Body weight is measured in kg (measured by the Weight Tronix scale), density is derived from BOD POD, and TBW is measured in kg. FFM will be calculated as body weight minus FM.

#### Energy Expenditure

EE will be measured during the last day of the 3 inpatient stays (presurgery, 3 months, and 12 months postsurgery). Height, weight, blood pressure, pulse, and temperature measurements will be taken, and an intravenous catheter will be inserted. At 8:00 a.m., subjects will be placed under the hood of the metabolic cart (Parvo Medics System, True Max 2400) [66] and rest for 30 minutes. Following the resting period, REE will be measured for 30 minutes and baseline blood samples will be taken. Subsequently, all subjects will consume an isocaloric liquid meal (Boost High Protein) over 10 minutes. Subjects will receive acetaminophen with the meal to measure gastric emptying. Following meal administration, DIT (amount of EE above the REE rate due to the cost of processing food for use and storage) will be measured for 4 hours. Blood samples and questionnaire measurements will be collected at –15, 0, 15, 30, 60, 90, 120, and 180 minutes to measure hormonal and perceived satiety. REE will be calculated as an average of the last 15-20 minutes of each measurement period if values have reached steady state, defined as <10% fluctuation in minute ventilation and oxygen consumption and <5% fluctuation in respiratory quotient. DIT will be calculated by measuring the area under the curve of postprandial metabolic rate, above extrapolated baseline REE, for each time period [67]. Baseline and postprandial CCK, GLP-1, ghrelin, and PYY gut peptide concentrations will also be measured during the meal test.

#### Satiety

Subjective and objective assessments of satiety will be collected before the surgery, at 3 months, and at 12 months postsurgery, during the meal test on the last day of the inpatient stay, using 2 different approaches: VASs, and measurements of hormonal signals of hunger and satiety.

##### Visual Analog Scales

Participants will rate their feelings on the following questions by means of a mark on 100-mm line VASs: “How hungry are you?”, “How full are you?”, “How much stomach discomfort do you feel?”, “How thirsty are you?”, and “How much anxiety and nervousness do you feel?” The scale will be anchored at the low end with the lowest intensity feelings (eg, *not at all*), and with opposing terms at the high end (eg, *most imaginable*), as previously described in the literature [68].

##### Measurements of Hormonal Signals of Satiety

Subjects will be instructed to consume the test meal within 15 minutes. Calorie intake and nutrient distribution of the meal (Boost High Protein) will be as follows: calories, 240; protein, 15 g; carbohydrates, 33 g; fat, 6 g; sodium, 200 mg; potassium, 400 mg, fiber 0 g. Patients will be able to choose between vanilla and chocolate flavors., An intravenous catheter will be inserted at 7:00 a.m. on the day of the experiment and blood will be drawn at before the meal, and at 15, 30, 60, 90, 120, and 180 minutes after the meal to measure hormonal signals of satiety. Blood samples will be collected in ethylenediaminetetraacetic acid tubes with added aprotinin (500 kallekrein inhibitory units per mL of blood) and dipeptidyl peptidase-4 inhibitor (10 μl/mL of blood; Millipore Research), and stored at –70° Celsius. Plasma concentrations of PYY, CCK, GLP-1, ghrelin, and insulin will be determined by radioimmune assay, and glucose concentration will be determined using the glucose oxidase method with an Analox glucose analyzer (Analox Instruments, Lunenburg, MA). Serum acetaminophen levels will be measured using an enzyme-linked immunosorbent assay (Abbot Laboratories, Chicago, IL).

##### Measure of Food Reward

Food reward is considered a strong eating drive that could override satiety, and will be assessed by the reward-based eating drive scale [69]. This 9-question screening tool will be completed by the participant during the inpatient stay before surgery, at 3 months, and at 12 months postsurgery.

#### Metabolomics—Branched Chain Amino Acids

Fasted blood samples obtained prior to the meal test will be used to measure circulating BCAAs by mass spectrometry, as previously described [70]. BCAAs will be measured during the inpatient stay before surgery, at 3 months, and at 12 months postsurgery.

#### Insulin Sensitivity

Insulin sensitivity will be measured by the Matsuda index during the meal test as follows: 10,000/(fasting glucose × fasting insulin × mean glucose from from 0 to 180 min) × mean insulin from from 0 to 180 min)^0.5 [71].

#### Physical Activity

There will be no physical activity intervention in this study. A measure of the free-living physical activity will be obtained using the ActiGraph, a wireless activity monitor that will provide 168 continuous hours (1 week) of measurement before surgery, at 3 months, and at 12 months postsurgery.

#### Vitamins, Minerals, Prealbumin, and Albumin Levels

Laboratory assessments will be obtained as part of the regular blood tests taken before surgery, at 3 months, and at 12 months postsurgery, following the clinical practice guidelines for the evaluation of the nutritional status in the Bariatric Clinic.

#### Food Record

Dietary intake evaluation during the outpatient phase of the study will be performed as part of each nutritional follow-up. Food and beverage intake will be assessed using either a 7-day (during the preceding diet and the inpatient stay) or 4-day (regular dietary evaluations) throughout the study. As described above, the PI goal will be accomplished for each group (HPS-G and SPS-G) by using the specific PS resources provided. During the screening phase of the study, all subjects will attend 2 training sessions delivered by a Registered Dietitian, in which they will be instructed on how to record their food intake and include at least 1 weekend day when recording. This information will be analyzed with NDSR.

### Sample Size Calculations

Sample size was calculated based on anticipated changes in REE during weight loss using data from published literature [72] and changes in BCAAs from our previous study [45]. Baba et al [72] studied changes in REE after high protein (n=7) versus low protein (n=6) weight loss diets. Weight loss was significantly higher in the high protein group (8.3 kg, SD 0.7) compared to the low protein (6.0 kg, SD 0.6). Change in REE was –132.3 kcal/day (SD 51.0) in the high protein group and –384.3 kcal/day (SD 84.6) in the low protein group. Assuming a Cronbach alpha of 0.05 and 0.80 (80%) power we would need to recruit 16 participants to each study group. Laferrère et al [45] showed that for a matched amount of weight loss (10 kg) in GBP (n=10) versus diet-induced weight loss (n=11), BCAA changes were –176.4 (SD 96.6) and –57.6 (SD 99.3) in surgical and nonsurgical groups, respectively. Assuming a Cronbach alpha of 0.05 and 0.80 (80%) power we would need to recruit 22 participants to each study group. Therefore, accounting for an attrition rate of 30% after 1 year, we will enroll a total of 28 subjects per group.

### Statistical Analyses

Data will be analyzed using the SPSS statistical program (IBM Software; Armonk, NY). For most study aims, a repeated measures design will be used to examine the trajectory of changes in subjects between HPS-G and SPS-G groups regarding BC, EE, NB, satiety, and circulating BCAAs, from presurgery to 3-month and 12-month postsurgery levels. Nonlinear mixed model regression (SAS PROC NLMIXED) will be used for the actual analyses since these outcomes are not likely to be normally distributed. In addition to overall tests for differences between groups (GBP and SG, and HPS-G vs SPS-G), differences over time (presurgery, 3 months, and 12 months postsurgery), and group per time interactions. Other factors will be explored as covariates to determine possible explanatory factors for significant differences between presurgery and 3-month and 12-month postsurgery levels. Significance levels will be adjusted based on the total number of comparisons being carried out, using a Bonferroni correction. Secondary outcomes will be analyzed using a similar repeated measures design with either mixed model regression or nonlinear mixed model regression where appropriate. No adjustment for the number of comparisons will be made in the case of the secondary outcomes (ie, Cronbach alpha will be 0.05 for all comparisons).

### Ethics

This proposal has been approved by the Institutional Review Board of Mount Sinai St Luke’s Hospital and Columbia University. Voluntary written informed consent will be obtained from each participant prior to enrollment.

## Discussion

The proposed study will determine the effect of 2 different levels of dietary PI (HPS-G and SPS-G) after SG and GBP on the NB, BC, EE, hormonal and perceived satiety, plasma levels of BCAAs, and insulin sensitivity, and feasibility of PS up to 1 year after BS. In addition, the analysis of energy excreted in feces will aid in the understanding of how much malabsorption exists in the 2 procedures studied (GBP and SG). Satiety during a liquid test meal will be assessed and its possible mediators, gastrointestinal hormones released, and gastric emptying rate will also be determined.

Two nonrandomized studies demonstrated that higher levels of PI (>60 g/day) were related to higher excess weight loss 6 months after BS [18] and at 3, 6, and 12 months postsurgery [37]. Other studies have failed to observe a significant association between PI and excess weight loss [28,35,73]. PI was positively associated with LBM retention in 3 nonrandomized studies [5,17,28], although this association was not found in other studies up to 1 year after BS [35,37]. The same authors also failed to observe a relationship between PI and REE [35,37]. The relationship between PI levels and gastrointestinal hormones needs to be explored more thoroughly. One RCT failed to observe a relationship between PI and GLP-1 or ghrelin [38]. The relationship between BCAA circulation levels and glucose homeostasis after BS also needs further attention. Elevated circulating BCAAs are associated with obesity and T2DM. Comprehensive metabolic profiling of obese versus lean human subjects revealed a BCAA metabolic signature, marked by increased circulating levels of BCAAs as well as products of BCAA catabolism [73]. The reason that circulating BCAA levels are elevated in obesity is still unclear. The mechanisms responsible for the decrease in BCAA serum levels with weight loss [47] or BS [46,74,75] are still being studied. Supplementation of a high fat diet with BCAAs in rats [76], or infusion of AAs in humans [76], results in IR. A recent epidemiological study reported that elevated plasma levels of essential AAs, including BCAAs, phenylalanine, and tyrosine in healthy individuals predicted a 5-fold increase in the risk of developing T2DM [77]. To our knowledge, there are no intervention studies that address the impact of PI on BCAA serum levels after GBP or SG. Lower levels of PI seem to have a positive effect on glucose homeostasis, while sustained low circulating levels of BCAAs may have a negative impact on protein synthesis and the integrity of the skeletal muscle mass during weight loss [78]. Plasma leucine concentration has been shown to correlate with skeletal muscle protein synthesis [53], so the metabolomic study of BCAAs during the high protein meal test will contribute to the study of AA kinetics after massive weight loss induced by BS. Measuring the NB during a negative energy balance will provide an important means of understanding absorption and bioavailability of nitrogen.

Dietary guidelines, including PS after BS, are still under discussion since the levels of evidence of their recommendations are C or D [24]. As previously detailed, the relationship between dietary PI and the various outcome variables that will be measured in this proposed study are not well established, and the available literature is contradictory. Dietary protein plays an important role in weight loss and obesity-related comorbidities, such as diabetes [79]. BS is highly popular, making the proposed work relevant, and the study will help to clarify the relationship between PI and BS outcomes by addressing some of the existing gaps in the scientific literature.

## Abbreviations

AA: amino acid
BC: body composition
BCAA: branched chain amino acid
BS: bariatric surgery
CCK: cholecystokinin
CRR: Clinical Research Resource
D2O: deuterium oxide
DIT: diet-induced thermogenesis
EE: energy expenditure
FFM: fat-free mass
FM: fat mass
GBP: gastric bypass
GLP-1: glucagon-like peptide 1
HPS-G: high protein supplementation group
IBW: ideal body weight
IR: insulin resistance
kcal: kilocalorie
LBM: lean body mass
NB: nitrogen balance
NDSR: Nutrition Data System for Research
PEG: polyethylene glycol
PI: protein intake
PS: protein supplementation
PYY: peptide YY
RCT: randomized controlled trial
REE: resting energy expenditure
SD: standard deviation
SG: sleeve gastrectomy
SPS-G: standard protein supplementation group
T2DM: type 2 diabetes mellitus
TBW: total body water
VAS: visual analog scale
